# The Role of Recipients’ Inter-Group Relations and Routine Behaviors in the Development of Children’s Sharing Behavior

**DOI:** 10.3390/bs15030254

**Published:** 2025-02-23

**Authors:** Hang Liu, Zhengmei Ma, Shiyin Chen, Lijie Zhang, Lu Wang

**Affiliations:** 1 Faculty of Education, Northeast Normal University, Changchun 130024, China; zwl@xjau.edu.cn (Z.M.); chenshiyin@nenu.edu.cn (S.C.); zhanglj426@nenu.edu.cn (L.Z.); wangl763@snnu.edu.cn (L.W.); 2 Urumqi Hongqi Kindergarten, Urumqi 830000, China; 3 Faculty of Education, Shaanxi Normal University, Xi’an 710062, China

**Keywords:** children, share, routine behaviors, inter-group relations, moral judgments

## Abstract

Sharing with others is an important prosocial behavior, which is widely developed in early childhood. Numerous studies have demonstrated that single characteristics (routine behaviors or inter-group relations) of the recipient impact children’s sharing behavior. However, there is still a lack of understanding of the factors that influence sharing decisions in children with dual characteristics. In Experiment 1, 112 children aged 4–6 years (*M_age_* = 5.55, girls account for 50%) participated in a sticker-sharing task, and the results indicated that older children (aged 5 and 6 years) were more willing to share and shared more stickers. Additionally, children shared more stickers with recipients who exhibited good routine behaviors compared to those who had poor behaviors. In total, 134 children aged 4–6 years (*M_age_* = 5.47, girls account for 50.1%) participated in Experiment 2. The results indicated that children share more stickers with a friend than with a stranger. When recipients possessed dual characteristics, they shared more stickers with a stranger who had good routine behaviors than with a friend who had poor ones. Therefore, children are selective in their sharing, and when confronted with a recipient with dual characteristics, children prioritize the recipient’s prior performance of moral norms over interpersonal distance.

## 1. Introduction

Sharing resources with others is a crucial prosocial behavior (Markovits et al., 2003; Steinbeis & Over, 2017). It initially appears in the second year of life, and it has been observed that generosity increases with age (House et al., 2013). Children aged 3–4 years already exhibit a tendency to share, especially when resources are earned by working collaboratively with others (Hamann et al., 2011) or when explicit communicative cues related to the recipients’ desires and needs are provided (Wu & Su, 2014). Around the age of 5, children begin to share resources with their peers equally and spontaneously (Rao & Stewart, 1999; Rochat et al., 2009). Learning to share with others helps children overcome egocentric tendencies (Fehr et al., 2008; House et al., 2013) and establish good peer relationships (Aknin et al., 2012; Gebauer et al., 2008; Paulus et al., 2015). In addition, sharing behaviors can predict children’s subsequent social adjustment (Crick, 1996) and academic achievement (Caprara et al., 2000).

In general, children’s sharing is characterized by selectivity, tending to share more with morally praiseworthy people than with others who violate morality (Warneken et al., 2011). Kenward and Dahl (2011) conducted a study in which 4.5-year-olds were more likely to share cookies with puppets who had previously been helpful than with those who had been violent or unhelpful. Similarly, Malti et al. (2016) discovered that 4-year-olds reward others for past moral behaviors, such as through sharing, even if it is costly for themselves. This shows that the recipient’s prior moral performance influences their sharing decisions, in terms of who they share with and how much or how little to share. In this context, previous studies have focused more on the prior prosocial behavior of the recipient; therefore how other moral factors (e.g., social norms) are taken into account by children when sharing has not yet been confirmed by current studies. Indeed, preschoolers already show an understanding of norms (Rakoczy et al., 2008); by the age of 3, children can assess norm violations by others (third parties) (Darley & Shultz, 1990; Ingram & Bering, 2010; Rizzo et al., 2018) and make behavioral decisions accordingly (Kenward & Dahl, 2011). Therefore, this study aims to investigate whether children share based on the prior performance of the person they are sharing with in terms of social norms.

Simultaneously, children rely on the relationships they have with recipients when they share with others (Paulus, 2016; Vonk et al., 2020; Yu et al., 2016). That is, children tend to share more with members of their in-group, such as friends (Olson & Spelke, 2008; Schmidt et al., 2012), than with strangers or disliked peers (Fehr et al., 2008). What remains controversial, however, is when the in-group tendency to share emerges. Some of the research confirms that children as young as 4 show in-group sharing tendencies. Moore’s (2009) findings indicated that 4-year-olds were more likely to share their stickers with friends, even if it meant giving away more, than with nonfriends or strangers. Similarly, a set of findings indicated that children preferred their good friends in sharing situations, with 4- to 5-year-olds sharing goods with friends at a higher rate than with strangers and nonfriends (Birch & Billman, 1986; Malti et al., 2016; McGuigan et al., 2016; Paulus & Moore, 2014; Paulus et al., 2015; Vaish et al., 2018); however, other researchers have suggested that children aged 5 and older are more likely to be influenced by inter-group relations during the sharing (Lu & Chang, 2016; Yu et al., 2016). The evidence on the age at which children are selective about who they share with and how this difference develops in early childhood remains mixed, and further research is urgently needed.

Interestingly, the reasons for children’s prosocial behaviors exhibiting in-group tendencies may be complex, and one reason is related to the potential moral label that children give to in-group members (Zhang et al., 2021). Several studies have shown that children tend to view in-group members as more likely to conform to social norms, while out-group members are perceived as more likely to violate them (Abrams et al., 2008; Aguilar et al., 2013; Chalik & Rhodes, 2020; Körner & Volk, 2014). The findings are probably due to the fact that when children evaluate the social norms of others from a third-party perspective, psychological distance varies with social distance from others, resulting in different level representations, which further affects moral judgments (Baron & Dunham, 2015; Zhang et al., 2021, 2024); however, a friend may not always follow certain rules, while a stranger may adhere to classroom discipline despite poor situations in real life, so what choices will children make when sharing in this situation? Are their choices more reliant on a moral code or on weighing the concerns of the group? There is limited knowledge of how children make complex sharing decisions for recipients with multiple characteristics.

In addition, previous studies have discussed children’s sharing behaviors primarily in virtual situations (Smetana et al., 2003; Yu et al., 2016), which are often detached from the realities of children’s lives, making it difficult to examine their spontaneous sharing in their daily lives. Indeed, children’s knowledge of morality and traditional norms originates from their everyday social experiences (Dahl & Freda, 2017). Therefore, before truly understanding social norms, children first understand the rules of daily life in kindergarten and judge and evaluate the behaviors of recipients mostly in terms of kindergarten norms of behavior. Therefore, the design of the situation in sharing tests is crucial. This study innovatively designed four types of situations (life, learning, activity, and communication) related to children’s lives in kindergarten, in which we examined the effects of the recipient’s behavioral performance and potential relationships with the children on the children’s sharing.

On the basis of the above background, this study proposes the following hypotheses: (1) as children grow older, they tend to exhibit more sharing behaviors; (2) when assessing potential recipients for sticker sharing, children prioritize those with good routine behavior over those who behave poorly; (3) children are more likely to share a greater number of stickers with friends compared to those belonging to strangers; and (4) when recipients have multiple characteristics, children will demonstrate greater sharing behavior with a stranger who exhibits good routine behavior rather than with a friend who exhibits poor routine behavior.

## 2. Experiment 1

### 2.1. Method

#### 2.1.1. Participants

Children were recruited randomly from five kindergartens in Lanzhou, a city in Northwest China. A total of 115 children aged between 4 and 6 years participated, and the majority hailed from middle-class families. Three children were excluded because they could not judge the routine behaviors of the recipient. The final sample included 112 children from three age groups: 4-year-olds (*N* = 35, *M*_age_ = 4.55, *SD* = 0.32, girls account for 48.9%), 5-year-olds (*N* = 37, *M*_age_ = 5.47, *SD* = 0.28, girls account for 41.7%), and 6-year-olds (*N* = 40, *M*_age_ = 6.50, *SD* = 0.26, girls account for 51.2%). Data were collected in September 2022, and informed consent was obtained from all caregivers.

#### 2.1.2. Design

A 4 × 2 × 3 mixed-model design was utilized in Experiment 1 with situation (life, learning, activity, communication) and routine behaviors (good, poor) as the within-participant variables and with age (4-, 5-, or 6-year-olds) as the between-participant variable. In total, 112 children were tested in each condition, which ensured sufficient statistical power. The sequence of 8 trials was presented to all children in a random order.

#### 2.1.3. Materials

The items to be shared were brightly colored stickers. The experimenter randomly selected 10 stickers from the prepared stickers for participants to choose. Stickers have been successfully used in previous studies, and a strong incentive effect on children has been confirmed (Bruner & Goodman, 1947; Jensen et al., 2025). Furthermore, we used white and pink envelopes as the sticker containers.

**Figure 1:**
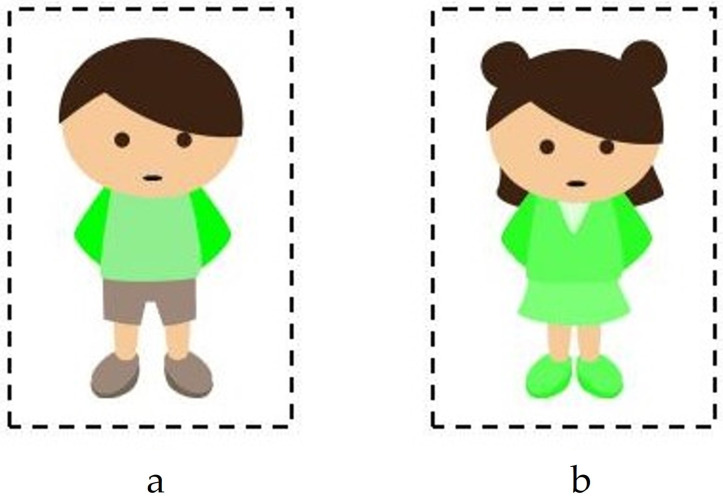
Diagram of the recipients Notes. The cartoon labeled (**a**) is intended for the boy’s viewing, while the cartoon marked (**b**) is designated for the girl’s perusal.

In addition, children were presented with 1.3 × 2.5 cm cartoon figure images to represent the recipient which matched the sex of children, and the images depicted a neutral mood (see Figure 1).

#### 2.1.4. Procedure and Scoring

The sticker-sharing task was used to examine the sharing behaviors of children in various situations when recipients exhibited good/poor routine behavior. Children were asked if they would like to participate and were then individually tested in a separate room of kindergarten by an experimenter, completing the sticker preference test and sharing test in turn.

(1) Sticker preference test

First, the child selected 8 favorite stickers from among the 10 prepared stickers (the stickers used the same material to avoid the influence of the stickers themselves). As demonstrated in the test, children of all ages selected their stickers with great care and treasured the stickers more after being told that the stickers belonged to them. Following their selection, the experimenter asked the child “Do you like your stickers?” All children affirmed forcefully that they liked their stickers.

(2) Sharing tests

Next, children were invited to judge the routine behaviors of the recipient. Once children answered correctly, they were guided to share their stickers with the recipient. Each child was asked to complete the sharing task in four situations (learning, life, activity, and communication).

Taking the life situation as an example, in which the experimenter described the recipient: “This child can eat meals attentively in the kindergarten, does not pick at food, does not waste food, and likes to wash his or her hands before and after meals”. The experimenter subsequently asked the child the following: (1) “Do you think he or she performs well in kindergarten?” If the child answered “yes”, the child was considered able to correctly judge moral behavior. The child was subsequently asked the following questions: (2) “This child thinks these stickers are pretty. Would you like to share these stickers with him or her?” (the experimenter pointed to the stickers owned by the child). If the child answered “yes”, he or she was invited to allocate the stickers and was prompted to put stickers for himself or herself in a white envelope and stickers for the recipient in a pink envelope. During their distribution, the experimenter reminded the child to freely allocate stickers, and no one knew the results of his or her distribution. By that time, the experimenter had turned around to perform other tasks to avoid interference from eye contact with the child. After all the tasks were finished, children were asked to leave the room with the white envelope (if they did not share, they would leave with all the stickers).

Based on the following situations (see Table 1), the number of stickers shared with the recipient in each situation was scored (a total of 8 stickers), the score in each situation ranged from 0 to 8.

#### 2.1.5. Data Analysis

SPSS 26.0 was used for the data analysis, and three-way repeated measures analysis of variance (ANOVA) was used to investigate the influence of the recipient’s routine behaviors on the child’s sharing behavior in various situations.

### 2.2. Results

Three-way ANOVA was repeatedly used to analyze children’s age, recipient routine behaviors and situation to test the influence on children’s sharing behaviors. The results revealed a large main effect of age [*F*(2, 109) = 38.384, *p* < 0.001, *η_p_*^2^ = 0.413]. A post hoc test revealed that the sharing scores of the 5-year-olds and 6-year-olds were significantly higher than those of the 4-year-olds, and there was no significant difference between the 6-year-olds and the 5-year-olds. The main effect of recipient routine behaviors was large [*F*(1, 109) = 758.115, *p* < 0.001, *η_p_*^2^ = 0.874]. Participants shared more stickers with those who had good routine behavior than with those with poor routine behavior. The main effect of the situations was also moderate [*F*(3, 107) = 4.341, *p* < 0.01, *η_p_*^2^ = 0.109]. A post hoc test revealed that the sharing scores in the learning, activity, and communication situations were significantly higher than those in the life situation. In addition, other effects were not significant.

The interaction effect between age and recipient’s routine behaviors was moderate [*F*(2, 109) = 8.511, *p* < 0.001, *η_p_*^2^ = 0.135]. Simple effect analysis showed that when the recipient had good routine behavior, the effect of age was large [*F*(2, 109) = 17.724, *p* < 0.001, *η_p_*^2^ = 0.245]. Specifically, the differences between the 6-year-olds and 4-year-olds were significant, and differences were also found between 5-year-olds and 4-year-olds. However, there was no significant difference between the 6-year-olds and the 5-year-olds. When the recipient had poor routine behavior, the effect of age was also large [*F*(2, 109) = 82.544, *p* < 0.001, *η_p_*^2^ = 0.602]. Specifically, the differences between the 6-year-olds and 5-year-olds were significant, and differences were found between the 6-year-olds and 4-year-olds and between the 5-year-olds and 4-year-olds. At the same time, in the three age groups, children gave more stickers to recipients with good routine behavior than to recipients with poor routine behavior [6-year-olds: *F*(1, 109) = 231.981, *p* < 0.001; *η_p_*^2^ = 0.680; 5-year-old group: *F*(1, 109) = 388.347, *p* < 0.001, *η_p_*^2^ = 0.781; 4-year-old group: *F*(1, 109) = 170.013, *p* < 0.001, *η_p_*^2^ = 0.609]. The results suggest that, in general, 4- to 6-year-olds take the recipient’s social norm behavior into account when sharing with others.

The interaction effect between the recipient’s routine behaviors and situation was large [*F*(3, 107) = 7.304, *p* < 0.001, *η_p_*^2^ = 0.170]. Simple effect analysis showed that when the recipient had good routine behaviors, the effect of the situations was large [*F*(3, 107) = 6.287, *p* < 0.01, *η_p_*^2^ = 0.150]. Multiple comparisons revealed that children shared more stickers with recipients in learning, activity, and communication situations than in life situations and significantly more in activity situations than in learning situations. When the recipient had poor routine behavior, the effect of the situations was also statistically significant, with a moderate effect size [*F*(3, 107) = 3.108, *p* < 0.05, *η_p_*^2^= 0.08], and multiple comparisons showed that the children shared more stickers with the recipient in the life situation than in the learning and activity situations. In all situations, the effect of routine behaviors was large [learning: *F*(1, 109) = 258.181, *p* < 0.001, *η_p_*^2^ = 0.723; life: *F*(1, 109) = 198.231, *p* < 0.001, *η_p_*^2^ = 0.645; activity: *F*(1, 109) = 343.848, *p* < 0.001, *η_p_*^2^ = 0.759; communication: *F*(1, 109) = 208.057, *p* < 0.001, *η_p_*^2^ = 0.656]. In addition, in all the situations, children always shared more stickers with recipients who had good routine behavior than with those who had poor routine behavior (see Table 2).

### 2.3. Discussion

In Experiment 1, we explored the age-related characteristics of children’s sharing behaviors and examined the impact of recipients’ routine behaviors on children’s sharing in various situations. The results showed that as children grow older, they tend to exhibit more sharing behaviors, and the age of 5 is a critical stage for the development of sharing in children. This developmental trend is consistent with previous research findings (Benenson et al., 2007; Blake & Rand, 2010; Fehr et al., 2008; Rochat et al., 2009).

Moreover, we confirmed that recipients’ routine behaviors impact children’s sharing, which means children tended to share more stickers with recipients who had good routine behavior than with those with poor one, and these results are consistent with previous findings (Malti et al., 2016; Olson & Spelke, 2008). These studies suggest that children’s sharing is selective and that they take the moral behaviors of the recipients into account when sharing. In particular, these results have been confirmed in various situations (life, communication, learning, and activity), which indicates that the impact of recipients’ routine behaviors on children’s sharing is consistent across situations. Further analysis revealed that, in the activity situation, the average deviation in the number of stickers shared with the recipients for different valences of routine behaviors was the largest, while the difference was the smallest in the life situations. This finding suggests that children are most sensitive to the routine behaviors of recipients during the activity situation.

In addition to the recipient’s routine behaviors, inter-group relations are a recipient characteristic that affects children’s sharing. Previous research has shown that children tend to share more with members of their in-group (Olson & Spelke, 2008; Schmidt et al., 2012) than with strangers or disliked peers (Fehr et al., 2008). In Experiment 2, we comprehensively explored how children share with recipients who have dual characteristics. Since Experiment 1 confirmed the cross-situation consistency of children’s sharing, we did not investigate the impact of situations in Experiment 2. In Experiment 2, we selected the activity situations, which are relatively sensitive to children, to examine the impact of the recipient’s routine behaviors and group identity on children’s sharing.

## 3. Experiment 2

### 3.1. Method

#### 3.1.1. Participants

Children were recruited randomly from 5 kindergartens in Lanzhou, a city in Northwest China. Importantly, the children who participated in Experiment 2 did not participate in Experiment 1. A total of 140 children aged between 4 and 6 years participated. Six children were excluded because they could not judge the routine behaviors of the recipient. The final sample consisted of 134 children from three age groups: 4-year-olds (*N* = 45, *M*_age_ = 4.58, *SD* = 0.27, girls account for 48.9%), 5-year-olds (*N* = 48, *M*_age_ = 5.47, *SD* = 0.31, girls account for 41.7%), and 6-year-olds (*N* = 41, *M*_age_ = 6.46, SD = 0.30, girls account for 51.2%). Data were collected in September 2022, and informed consent was obtained from all caregivers.

#### 3.1.2. Design

A 2 × 2 × 3 mixed-model design was utilized in Experiment 2 with inter-group relations (in-group or out-group) and routine behaviors (good, poor) as the between-participant variables and with age (4-, 5-, or 6-year-olds) as the between-participant variable. In other words, 134 children were tested in each condition, which ensured sufficient statistical power. The sequence of 4 trials was presented to all children in a random order.

#### 3.1.3. Materials

**Figure 2:**
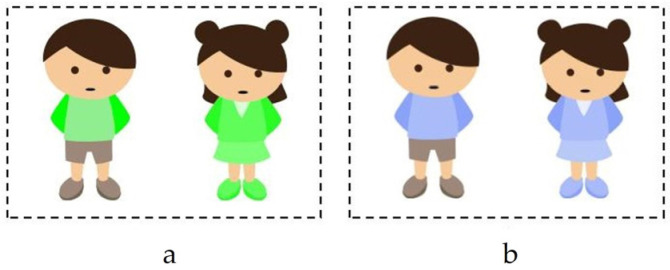
Diagram of the recipients (out-group and in-group). Notes. the green cartoon diagrams (**a**) represented strangers, while the blue diagrams (**b**) represented friends.

The sticker preference test involved the same materials as those used in Experiment 1. The images of the recipients were divided into two groups, an in-group and an out-group, with each group consisting of one boy and one girl cartoon diagram. The green cartoon diagram represented the out-group recipient, while the blue diagram represents the in-group recipient, with the same image format as in Experiment 1. During the experiment, the experimenter reminded the children to distinguish the inter-group relations of the characters according to their color (see Figure 2).

#### 3.1.4. Procedure and Scoring

The sticker-sharing task was used to investigate the characteristics of children’s sharing behaviors toward recipients with various inter-group memberships and routine behaviors. The experimental environment was the same as that in Experiment 1, and the children completed the sticker preference test and sharing tests sequentially. We replicated the procedure of Experiment 1 in the sticker preference test, with changes implemented in the sharing tests.

Before beginning, the experimenter asked the child who his or her friend was and then presented the in-group cartoon diagram (blue) as representing the friend. Later, the child was asked an understanding question: “Which of these two children represents your friend?”. Next, the experimenter described the recipients and questioned the child about the in-group recipient (the child’s friend) and the out-group recipient (a stranger), who was in the background of the activity situation. For example, for a recipient with good routine behavior, the child was asked “During classroom activities, your friend (or a stranger) takes great care of the things for the game and follows the rules of the game while playing outdoors. Do you think he or she did well?”. If the child judged the situation correctly, he or she was asked “your friend (or a stranger) also likes your stickers, would you like to share with him or her?”. If the child answered “yes”, he or she was prompted to put the stickers in pink envelopes (for others) or white envelopes (for himself or herself). The remaining procedures and settings are the same as in Experiment 1. The children were asked to leave the room after all the tasks were completed.

The scoring method was the same as that in Experiment 1.

### 3.2. Results

Three-way ANOVA was repeatedly used to analyze children’s age (4-, 5-, or 6-year-olds), recipients’ routine behaviors (good or poor), and inter-group relations (in-group or out-group) to test the influence on children’s sharing behaviors. The results revealed that the main effect of age was large [*F*(2, 131) = 71.932, *p* < 0.001, *η_p_*^2^ = 0.523]. Multiple comparisons revealed that the sharing scores of the 6-year-olds and 5-year-olds were significantly higher than those of the 4-year-olds, and there was no significant difference between the 6-year-olds and the 5-year-olds. The main effect of recipient routine behaviors was large [*F*(1, 131) = 868.036, *p* < 0.001, *η_p_*^2^ = 0.869]; children shared more stickers with recipients with good routine behavior than with those with poor routine behavior. The main effect of inter-group relations was large [*F*(1, 131) = 237.965, *p* < 0.001, *η_p_*^2^ = 0.645]; children shared more stickers with in-group recipients than with out-group recipients (see Table 3).

The interaction effect between age and the recipient’s routine behaviors was large [*F*(2, 131) = 23.865, *p* < 0.001, *η_p_*^2^ = 0.267]. Simple effect analysis showed that when the recipient had good routine behavior, the effect of age was large [*F*(2, 131) = 64.612, *p* < 0.001, *η_p_*^2^ = 0.497]. Multiple comparisons revealed that the sharing score was significantly higher for the 6-year-olds and 5-year-olds than for the 4-year-olds, and there was no significant difference between the 6-year-olds and the 5-year-olds. When the recipient had poor routine behavior, the effect of age was moderate [*F*(2, 131) = 9.180, *p* < 0.01, *η_p_*^2^ = 0.123]; the sharing scores of the 6-year-olds were significantly higher than those of the 5-year-olds and the 4-year-olds, and the sharing scores of the 5-year-olds were significantly higher than those of the 4-year-olds. At the same time, in the three age groups, children gave more stickers to the in-recipient group than to the out-group recipient [4-year-olds: *F*(1, 131) = 122.086, *p* < 0.001, *η_p_*^2^ = 0.482; 5-year-olds: *F*(1, 131) = 506.268, *p* < 0.001, *η_p_*^2^ = 0.794; 6-year-olds: *F*(1, 131) = 330.144, *p* < 0.001, *η_p_*^2^ = 0.716].

The interaction between the recipient’s routine behaviors and the inter-group relations was moderate [*F*(1, 131) = 17.378, *p* < 0.01, *η_p_*^2^ = 0.117]. Simple effect analysis showed that when the recipient had good routine behavior, children shared more stickers with in-group recipients than with out-group recipients [*F*(1, 131) = 181.686, *p* < 0.001, *η_p_*^2^ = 0.58]; when the recipient had poor routine behavior, children shared more stickers with in-group recipients than with out-group recipients [*F*(1, 131) = 77.790, *p* < 0.001, *η_p_*^2^ = 0.373]. When sharing with friends, the children gave more stickers to the recipients who behaved well than to the recipients who behaved badly [*F*(1, 131) = 583.358, *p* < 0.001, *η_p_*^2^ = 0.817]. When sharing with strangers, the children gave more stickers to the recipients who behaved well than to the recipients who behaved badly [*F*(1, 131) = 744.500, *p* < 0.001, *η_p_*^2^ = 0.850]. A paired-samples t test revealed that, compared with in-group recipients with poor routine behavior, children shared more stickers with out-group recipients with good routine behavior [*t* (133) = −14.245, *p* < 0.001, Cohen’s d = 0.316].

**Figure 3:**
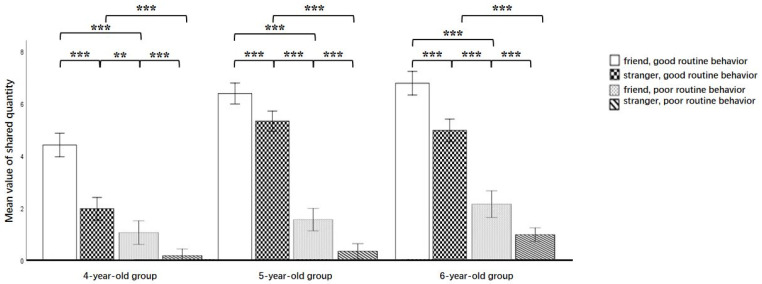
Number of stickers shared by children on different inter-group relations and routine behaviors (out of 8). Notes: ** *p* < 0.01, *** *p* < 0.001.

The interaction effects among age, recipient’s routine behaviors, and inter-group relations were moderate [*F*(2, 131) = 9.930, *p* < 0.01, *η_p_*^2^ = 0.132]. Simple effect analysis revealed that when the in-group recipient exhibited good routine behavior, the effect of age was large [*F*(2, 131) = 31.309, *p* < 0.001, *η_p_*^2^ = 0.323]. The sharing scores of the 5-year-olds and 6-year-olds were significantly higher than those of the 4-year-olds; moreover, there was no significant difference between the scores of the 6-year-olds and the 5-year-olds. When the in-group recipient had poor routine behavior, the effect of age was moderate [*F*(2, 131) = 5.193, *p* < 0.05, *η_p_*^2^ = 0.073]. Similarly, the sharing scores for the 5-year-olds and 6-year-olds were significantly higher than those for the 4-year-olds, and there was no significant difference between the 6-year-olds and the 5-year-olds. When the out-group recipient exhibited good routine behavior, the effect of age was large [*F*(2, 131) = 70.434, *p* < 0.001, *η_p_*^2^ = 0.518]; the sharing scores of the 5-year-olds and 6-year-olds were significantly higher than those of the 4-year-olds, and there was no significant difference between the 6-year-olds and the 5-year-olds. When the out-group recipient exhibited poor routine behavior, the effect of age was still large [*F*(2, 131) = 11.459, *p* < 0.01, *η_p_*^2^ = 0.149], and the sharing scores of the 6-year-olds were significantly higher than those of the 5-year-olds and 4-year-olds; however, there was no significant difference between the 5-year-olds and the 4-year-olds. Among the three age groups, children gave more stickers to in-group recipients with good routine behavior than to those with poor routine behavior [6-year-olds: *F*(1, 131) = 222.324, *p* < 0.001, *η_p_*^2^ = 0.629; 5-year-old group: *F*(1, 131) = 275.534, *p* < 0.001, *η_p_*^2^ = 0.678; 4-year-old group: *F*(1, 131) = 114.109, *p* < 0.001, *η_p_*^2^ = 0.466]. At the same time, in the three age groups, children gave more stickers to the out-group recipients with good routine behavior than to those with poor routine behavior [6-year-olds: *F*(1, 131) = 282.475, *p* < 0.001, *η_p_*^2^ = 0.683; 5-year-old group: *F*(1, 131) = 543.494, *p* < 0.001, *η_p_*^2^ = 0.806; 4-year-old group: *F*(1, 131) = 64.890, *p* < 0.001, *η_p_*^2^ = 0.331]. A paired-samples test revealed that, compared with in-group recipients with poor routine behavior, children shared more stickers with out-group recipients with good routine behavior [4-year-olds: *t* (40) = −2.741, *p* < 0.01, Cohen’s d = 0.111); 5-year-olds: *t* (51) = −12.640, *p* < 0.001, Cohen’s d = 0.225; 6-year-olds: *t* (40) = −8.088, *p* < 0.001, Cohen’s d = 0.182] (see Figure 3).

### 3.3. Discussion

In Experiment 2, we simultaneously investigated the impact of age, the recipient’s routine behaviors, and inter-group relations on children’s sharing. In terms of age development patterns, Experiment 2 replicated the results of Experiment 1. Similarly, the results of Experiment 2 revealed that, compared to recipients with poorer routine behaviors, children tend to give more stickers to recipients with better routine behaviors. Additionally, Experiment 2 discussed the role of inter-group relations in children’s sharing and revealed that 4-year-olds, 5-year-olds, and 6-year-olds all shared more stickers with friends than with strangers. This finding suggests that, beginning at age 4, children exhibit an in-group bias when sharing, which is consistent with previous research findings (Olson & Spelke, 2008; Paulus, 2016; Schmidt et al., 2012; Vonk et al., 2020; Yu et al., 2016). The more significant finding was that children shared more stickers with strangers who exhibited better routine behaviors than with friends who exhibited poor routine behavior. This finding suggests that when recipients have dual characteristics, children place more emphasis on the recipient’s moral normative behaviors than on the psychological distance when sharing.

## 4. General Discussion

This study explored developmental differences in children’s resource sharing and the impact of recipients’ routine behaviors and inter-group relations. The findings suggest that children’s spontaneous sharing increases significantly with age, with the age of 5 being a critical period for acquiring generous sharing. Based on the analysis conducted, we concluded that Hypothesis 1 is supported. This indicates that beyond the age of five, children’s sharing behavior undergoes a transformation, evolving from a state of egocentrism to an intricate process of social decision-making (Baron & Dunham, 2015; Renno & Shutts, 2015). Such a shift signifies the attainment of maturity in young children’s social cognition and moral emotions. Consequently, it aids them in establishing a foundational groundwork for their future social adaptability (Chai et al., 2021). Older children (5 years and older) are more willing to share with others than younger children (4 years). This reaffirms the trend of individual sharing behaviors during the preschool years (Blake & Rand, 2010; Fehr et al., 2008; Rochat et al., 2009). In particular, in situations where recipients were routinely well behaved, children aged 5 years and older shared more than half of the average number of stickers (>5), whereas children in the 4-year-old group shared fewer than half of the total number of stickers on average (<4). Research suggests that sharing may reach a watershed at age 5. Children younger than 5 years tend to be more selfish (Benenson et al., 2007; Blake & Rand, 2010; Fehr et al., 2008; Rochat et al., 2009; Thompson et al., 1997; Wu & Su, 2014) and still have strong egocentric motives. However, children aged 5 and above tend to share more resources with others (Rao & Stewart, 1999; Rochat et al., 2009). On the one hand, children’s social cognitive abilities, such as their theory of mind abilities, may progressively develop and mature. This leads children to become increasingly concerned about the welfare of others (Fehr et al., 2008), which in turn strengthens their prosocial motivation (Lane et al., 2010; Ruffman et al., 2006; Sally & Hill, 2006; Walker, 2005). On the other hand, cultural factors may also play a role. Most of the age-consistent findings to date have been obtained from studies with Chinese children (Rao & Stewart, 1999; Rochat et al., 2009). In Eastern cultures that promote collectivist values, children may exhibit altruistic tendencies earlier (Wu & Su, 2014). Based on the human nature of subordination and imitation, altruistic behaviors are more socially normative when collectivism is emphasized in educational environment and public perceptions (Rochat et al., 2009). However, in this study, the cultural factors that underlie children’s motivations for sharing behavior have not been fully investigated and analyzed. Future research could focus on examining how children from diverse cultural backgrounds interpret the reasons for sharing. Through comparative analysis, the mechanisms by which cultural factors influence children’s motivations for sharing behavior can be explored in depth.

Our observations revealed that children tended to share more stickers with recipients who exhibited good routine behavior than with those who exhibited poor ones. Upon careful consideration of the evidence, we concluded that Hypothesis 2 is supported. These results are consistent with previous findings. Children are more likely to share with recipients who are morally deserving (Malti et al., 2016). In other words, according to their understanding of moral norm and motivated by the recipient’s previous moral behaviors, they share more resources with recipients of good moral behaviors such as those who are prosocial (Baumard et al., 2012; Kanngiesser & Warneken, 2012; Kenward & Dahl, 2011; House et al., 2020). This finding suggests that children’s understanding of moral norms is linked to their prosocial behaviors (Malti et al., 2010). Additionally, preschoolers can make moral judgments based on the actions of third parties, which can impact their own complex prosocial behaviors toward the actor (Kenward & Dahl, 2011). This highlights the strong correlation between moral judgments and prosocial behaviors at an early age. For instance, research has shown that infants as young as 6 and 10 months old tend to approach individuals who have helped others and avoid those who have hindered others (Hamlin et al., 2008). Additionally, 8-month-olds have been found to selectively respond positively to prosocial individuals and negatively to antisocial individuals (Hamlin & Wynn, 2011). However, they rely more on conscious activities than on perceptual activities to differentiate between prosocial and antisocial people in early childhood (Kenward & Dahl, 2011). The present study further showed that 5-year-old children consider the benefit of the recipient’s behavioral norms more than 4-year-old children when sharing with others. This finding suggests that starting at age 5, children share at least partially based on moral considerations (Cooley & Killen, 2015; Dahl & Waltzer, 2020), whereas 4-year-olds have a stronger normative sense of property and rights (Pesowski & Friedman, 2018; Schmidt et al., 2012).

This study investigated the impact of inter-group relations on children’s sharing behaviors. The results indicated that children shared more stickers with in-group members and fewer with out-group members. By analyzing the evidence, we have reached the conclusion that Hypothesis 3 is firmly supported by our findings. These findings are consistent with those of previous studies (Fehr et al., 2008; Malti et al., 2016; Moore, 2009; Paulus & Moore, 2014; Paulus et al., 2015). According to Essler et al. (2020), children’s sharing in early childhood is selective and influenced by the characteristics of the recipient group. From a social development perspective, individuals may be motivated to behave in ways that benefit their social group. This behavior can strengthen productive social bonds between individuals and contribute to the success of the group as a whole (Göette et al., 2006; Hailey & Olson, 2013; Trivers, 1971). This finding also supports the idea of social reasoning development (SRD), which suggests that in inter-group situations, developing children not only show concern for fairness but also weigh the concerns of the group when allocating resources (Essler et al., 2020; Killen et al., 2018; Rutland & Killen, 2017).

At the same time, we are more interested in the age at which children start to show group preferences. The results of this study show that children, whether they are 4, 5, or 6 years old, share more stickers with their friends than with strangers. This finding suggests that around the age of 4, an in-group preference emerges in children’s sharing. This finding is consistent with some of the findings of previous studies, such as Moore (2009), who found that 4.5- to 6-year-olds were more willing to give up valuable resources if they benefited a friend (as opposed to a stranger or a disliked peer). Other studies have also confirmed that around the age of 4, children begin to be more generous to their friends than to others (Buhrmester et al., 1992; Paulus & Moore, 2014; Yu et al., 2016), while expecting others to be more generous to their friends (Olson & Spelke, 2008). However, it has also been proposed that there is a developmental change in preschoolers’ sharing behaviors toward different groups, i.e., 5- and 6-year-olds are more inclined to share with their friends than with strangers than 4-year-olds are (Fehr et al., 2013). It has even been suggested that kinship tendencies in prosocial behaviors may not emerge at the end of preschool (Lu & Chang, 2016). The reason for this difference is related to children’s understanding of the concept of inter-group relations, where the distribution of resources between groups may be more complex than that between individuals, and group attention may influence children’s resource allocation (An et al., 2020).

This study also explored the joint role of recipients’ routine behaviors and inter-group relations in children ’s sharing behaviors. An important finding was that children shared more stickers with strangers with good routine behavior than with their friends with poor ones. After a detailed analysis of the collected data, we affirm that Hypothesis 4 is confirmed by our study. This finding suggests that children prioritize recipients’ prior moral performance when they have multiple characteristics and engage in more just sharing behaviors and decisions based on the recipient’s moral performance (Baumard et al., 2012; Kenward & Dahl, 2011). A plausible explanation is that as they age, children become adept at integrating conflicting motives such as selfish considerations, moral considerations, and group-based motives (Rutland & Killen, 2017), and in this process, their prosocial behaviors are more likely to act in accordance with moral norms than humane ones. In daily life, children may learn traditional norms from authority figures such as teachers and parents or by referring to existing rules (Blake et al., 2016; Chai et al., 2024). For instance, in Chinese kindergartens, well-behaved children are often praised and rewarded by their teachers. This teaches children that good behavior is deserving of recognition. Furthermore, according to the moral principle of indirect reciprocity, children comprehend that strangers who behave well are more likely to reciprocate than close people who routinely behave poorly (Levitt et al., 1985; Olson & Spelke, 2008). Meanwhile, the present study recorded the children’s sharing behaviors only, but failed to record the motivation of children’s sharing. This might have an impact on the interpretation of the results; for example, they may consider sharing as a “due” reward for the good behavior of the recipient. Future research should impose stricter controls on extraneous variables and delve deeper into the relevant content concerning motivation.

All in all, in terms of practical implications, our findings might help us to better understand how and when inter-group relations and moral judgments have a joint influence on children’s sharing behaviors, especially in real life. This suggests that adults should conform to the rules of children’s development when cultivating their sharing behavior, and guide children to share spontaneously when appropriate, such as when the recipient is a more moral person or a closer person. In this way, children are more likely to experience the joy of sharing, which promotes their development to higher levels, such as non-discriminatory fairness.

## 5. Conclusions

The present study provides new insights into the factors that may influence sharing behaviors in early childhood. (1) Spontaneous sharing by young children increases significantly with age and shows consistency across situations. Among these, the age of 5 is a critical period for young children to share generously. (2) Children tend to share with recipients who show good behavior or who belong to the members of their in-group when sharing. (3) Children are more inclined to share with recipients who are friends, as opposed to those who are strangers. (4) When recipients exhibit multiple characteristics, young children show a tendency to share with a well-behaved stranger over a friend who behaves poorly.

## Figures and Tables

**Table 1:** Description of the experimental context and instructions.

Situation	Routine Behavior	Description
Life	Good	This child does not pick at food or waste food, he/she eats meals attentively, and likes to wash hands before and after meals.
	Poor	This child picks at food and wastes food, he/she does not eat meals attentively, and does not wash hands before and after meals.
Learning	Good	This child listens to class carefully, and answers questions actively, strictly keeping classroom discipline.
	Poor	This child does not pay attention in class, and often interjects when other children are answering questions, usually disobeying classroom discipline.
Activity	Good	This child takes great care of the material for games in indoor activities and follows the rules of the game when playing outdoors.
	Poor	This child always destroys the material for games in indoor activities and breaks the rules of the game when playing outdoors.
Communication	Good	This child can actively greet the teacher and use polite language when requesting help and never fights or bullies others.
	Poor	This child never greets the teacher proactively, does not use polite language and often fights with other children and bullies them.

**Table 2:** Descriptive statistics of age, routine behaviors, and situations (*N* = 112).

Age	Routine Behavior	Situation	*M*	*SD*
4-year-olds	Good	Learning	3.62	2.64
		Life	2.70	1.88
		Activity	4.00	2.48
		Communication	3.81	2.70
	Poor	Learning	1.13	0.35
		Life	1.18	0.40
		Activity	1.00	0.00
		Communication	1.20	0.45
5-year-olds	Good	Learning	5.59	2.26
		Life	4.60	2.02
		Activity	5.10	2.28
		Communication	5.62	2.37
	Poor	Learning	1.24	0.56
		Life	1.25	0.59
		Activity	1.16	0.37
		Communication	2.12	1.13
6-year-olds	Good	Learning	5.05	2.24
		Life	4.38	2.06
		Activity	5.33	1.95
		Communication	5.20	2.43
	Poor	Learning	1.54	0.82
		Life	1.90	0.98
		Activity	1.58	0.87
		Communication	2.36	1.42

**Table 3:** Descriptive statistics of age, inter-group relations, and routine behaviors (*N* = 134).

Age	Inter-Group Relation	Routine Behavior	*M*	*SD*
4-year-olds	In-group	Good	4.42	0.23
		Poor	0.90	0.22
	Out-group	Good	2.12	0.22
		Poor	0.15	0.11
5-year-olds	In-group	Good	6.39	0.20
		Poor	1.54	0.19
	Out-group	Good	5.33	0.19
		Poor	0.25	0.10
6-year-olds	In-group	Good	6.78	0.23
		Poor	1.88	0.22
	Out-group	Good	4.98	0.22
		Poor	0.85	0.11

## Data Availability

The data presented in this study are available on request from the corresponding author.
